# Regulation of Vitamin C Accumulation for Improved Tomato Fruit Quality and Alleviation of Abiotic Stress

**DOI:** 10.3390/genes12050694

**Published:** 2021-05-06

**Authors:** Ifigeneia Mellidou, Athanasios Koukounaras, Stefanos Kostas, Efstathia Patelou, Angelos K. Kanellis

**Affiliations:** 1 Institute of Plant Breeding and Genetic Resources, Hao Elgo-Demeter, 57001 Thessaloniki, Greece; 2 Department of Horticulture, Aristotle University of Thessaloniki, 54124 Thessaloniki, Greece; thankou@agro.auth.gr (A.K.); skostas@agro.auth.gr (S.K.); 3 Laboratory of Pharmacognosy, Group of Biotechnology of Pharmaceutical Plants, Department of Pharmaceutical Sciences, Aristotle University of Thessaloniki, 54124 Thessaloniki, Greece; epatelou@yahoo.gr

**Keywords:** ascorbate, biofortification, environmental stimuli, plant stress, ethylene, genetic modifications, postharvest

## Abstract

Ascorbic acid (AsA) is an essential multifaceted phytonutrient for both the human diet and plant growth. Optimum levels of AsA accumulation combined with balanced redox homeostasis are required for normal plant development and defense response to adverse environmental stimuli. Notwithstanding its moderate AsA levels, tomatoes constitute a good source of vitamin C in the human diet. Therefore, the enhancement of AsA levels in tomato fruit attracts considerable attention, not only to improve its nutritional value but also to stimulate stress tolerance. Genetic regulation of AsA concentrations in plants can be achieved through the fine-tuning of biosynthetic, recycling, and transport mechanisms; it is also linked to changes in the whole fruit metabolism. Emerging evidence suggests that tomato synthesizes AsA mainly through the l-galactose pathway, but alternative pathways through d-galacturonate or *myo*-inositol, or seemingly unrelated transcription and regulatory factors, can be also relevant in certain developmental stages or in response to abiotic factors. Considering the recent advances in our understanding of AsA regulation in model and other non-model species, this review attempts to link the current consensus with novel technologies to provide a comprehensive strategy for AsA enhancement in tomatoes, without any detrimental effect on plant growth or fruit development.

## 1. Introduction

Vitamin C (vitC) or ascorbic acid (AsA) is one of the most abundant water-soluble antioxidant molecules, essential for aerobic life, presents in nearly all living organisms, including plants, animals, fungi, and protozoa [1,2,3,4]. The AsA biosynthetic pathways differ between plants, animals, and photosynthetic protozoa, whereas fungi are able to synthesize a 5C analog of AsA, namely d-erythroascorbate [5]. In the mammalian pathway, AsA is mainly synthesized in the liver, with the last step being catalyzed by the enzyme l-gulono-1,4-lactone oxidase (GuLO), a Flavin Adenine Dinucleotide (FAD)-linked enzyme related to the endoplasmic reticulum membrane. Humans, other primates, and a few other mammals including guinea pigs, and certain groups of bats and birds, have lost the ability to synthesize this low molecular weight molecule as a result of mutations in the coding sequence of GuLO [4,6]. This loss of capacity may serve as an evolutionary step towards the regulation of human cell redox homeostasis since the reaction catalyzed by GuLO in the biosynthetic pathway also generates hydrogen peroxide (H_2_O_2_), a toxic Reactive Oxygen Species (ROS) that could eliminate the advantage of AsA biosynthesis [7].

Several lines of evidence suggest that AsA, by scavenging ROS, can protect DNA, proteins, and lipids from oxidative damage in the human cells. Therefore, AsA has been involved in iron bioavailability and the inhibition of ferritin degradation, protecting from anemia, in the biosynthesis of many signaling peptides, in cytochrome P450 dependent hydroxylation, in collagen formation, while it also serves as a co-factor of hydroxylases [8]. Severe AsA deficiency may cause scurvy [9,10]. Due to its ability to modulate epigenome, AsA has been recently proposed as an effective anti-cancer molecule [11]. An average requirement of 90 mg/day for men and 80 mg/day for women, has been recommended by the European Food Safety Authority, but this recommended intake can be significantly higher for pregnant women or when adults are exposed to stress, smoking, or alcoholism, which cause a rapid decline in plasma ascorbate concentration (https://efsa.onlinelibrary.wiley.com/doi/pdf/10.2903/sp.efsa.2017.e15121, accessed on 5 May 2021). Although vitC-deficiency has declined throughout time, especially in developing countries, the recent fatal human coronavirus disease (named COVID-19), necessitates a diet rich in vitC for good health. In particular, vitC has been recently demonstrated to act beneficially not only as prophylaxis (at low doses) but also in cases of severe COVID-19, as vitC can reduce inflammatory mediators including interleukin-6 and endothelin-1, which drive pneumonia and respiratory failure in hypertensive and diabetic adult patients [12].

In spite of the fact that chemically synthesized AsA is nearly indistinguishable from the plant-derived one, fresh fruits and vegetables serve as the main source of vitC for humans [3]. Interactions with other phytonutrients, especially vitamin E, should be also taken into consideration when studying the bioavailability of ascorbate [6], although findings so far dictate whether bioavailability is higher or lower in plant-derived resources compared to synthetic supplements [8].

Due to its remarkable functions in plant growth and development, in plant abiotic stress responses, as well as its nutritional benefits in the human diet, genetic regulation of AsA in plant species with edible organs, and in particular tomato (*Solanum Lycopersicum* L.), has received considerable attention over the last years. Tomato is one of the most pronounced vegetable crops worldwide, being considered as a model species to investigate ripening and quality attributes in fruit species. Despite accumulating moderate levels of AsA in fruits, tomato has been employed extensively to study AsA metabolic pathways in fruits, probably due to its significance in the human diet, as well as its high levels of consumption [13,14,15].

Currently, commercial varieties and hybrids seem to possess lower amounts of AsA in many fruit crop species, including tomato, compared to wild relatives, probably as a joint result of the dilution effect and the optimum-devoid of stress-greenhouse growing conditions [3]. Conceptually, the enhancement of AsA in plants represents a strategic goal for improved human health. On the basis of these considerations, this review attempts to elucidate recent findings in the genetic factors underlying AsA accumulation in fruit species, focusing on tomato, for improved fruit quality and alleviation of abiotic stress.

## 2. Biosynthesis and Catabolism of Ascorbic Acid in Plants

**Figure 1:**
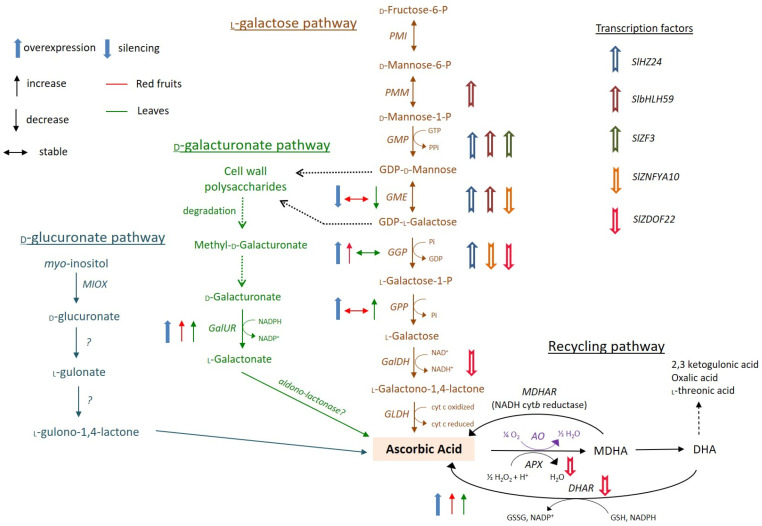
AsA metabolic pathways in tomato. The main biosynthetic pathway occurring via l-galactose is shown in brown, the d-galacturonate pathway in green, the d-glucuronate pathway in blue, and the AsA recycling pathway in black. Purple symbolizes a reaction taking place in the apoplast. Efficient manipulations of structural genes in either leaves (green) or fruits (red) via transgenic efforts are presented on the left of the reactions with blue arrows, followed by their impact on the AsA pool (increase, decrease, or stable). The fold change >1.5 (overexpression) or <0.5 (silencing) of the AsA contents in transgenic plants compared to wild-type plants was regarded as efficient manipulation. The full list of transgenic approaches is given in Table 1. Regulatory factors affecting positively or negatively the transcription of structural genes are also presented with arrows on the right of the reaction. PGI: Phosphoglucose Isomerase; PMI: Mannose-6-phosphate isomerase; PMM: Phosphomannomutase; GMP: GDP-d-mannose pyrophosphorylase; GME: GDP-d-mannose 3′5′ epimerase; GGP: GDP-l-galactose-phosphorylase; GPP: l-galactose-1-P phosphatase; GalDH: *L*-galactose dehydrogenase; GLDH: *L*-galactono-1,4-lactone dehydrogenase; GalUR: *D*-galacturonate reductase; MIOX: *myo*-inositol oxygenase; GuLO: *L*-gulono-1,4-lactone dehydrogenase; AO: ascorbate oxidase; APX: ascorbate peroxidase; MDHAR: monodehydro-ascorbate reductase; DHAR: dehydro-ascorbate reductase; MDHA: monodehydroascorbate; DHA: dehydroascorbate; GSH: glutathione; GSSG: oxidized glutathione.

In plants, AsA can be primarily synthesized via the so-called l-galactose pathway, as initially described by Wheeler et al. [1]. This main biosynthetic pathway consists of eight enzymatic steps (Figure 1), with the first steps prior to GDP-d-mannose involving cell wall polysaccharide precursors and glycoproteins [16]. In this route, d-fructose-6-P derived from d-glucose is initially converted to d-mannose-6-P via phosphomannose isomerase (PMI). In turn, d-mannose-6-P forms d-mannose-1-P via phosphomannomutase (PMM), and d-mannose-1-P forms GDP-d-mannose via GDP-d-mannose pyrophosphorylase (GMP). All the downstream reactions after this step are solely committed to AsA biosynthesis, starting with the conversion of GDP-d-mannose to GDP-l-galactose in a reaction catalyzed by GDP-d-mannose-3,5-epimerase (GME), an enzyme of the extended short-chain dehydratase/reductase protein family. This latter gene has been initially considered critical for the regulation of the AsA pool size in plant tissues [17,18], as it represents the intersection between the l-galactose and cell-wall biosynthesis related pathways [19]. Noteworthy, GDP-l-gulose can also be produced if GME catalyzes 5′ epimerization instead of 3′,5′ epimerization, but as this sugar has no particular function in plants, it has been suggested that it is directly channeled to AsA synthesis [6]. After this key step, GDP-l-galactose is transformed into l-galactose-1-P, l-galactose and l-galactono-1,4-lactone, in the reactions catalyzed by GDP-l-galactose-phosphorylase (GGP) encoded by *VTC2* and *VTC5* in *Arabidopsis* [20,21], by l-galactose-1-phosphate phosphatase (GPP) encoded by *VTC4* in *Arabidopsis* [22,23], and by the NAD-dependent l-galactose dehydrogenase (GalDH) [24], respectively. In fact, the reaction catalyzed by GGP serves as the first dedicated step to AsA biosynthesis, and the regulation of the pathway largely resides at this gene [5].

The *Arabidopsis vtc*-deficient mutants enabled the functional characterization of these intermediate steps of the main pathway. Considerable attention has been given to *GMP* with the identification of the well-characterized *Arabidopsis vtc1* mutants, which contain 30% less AsA compared to the wild-type (WT) plants [25]. On the other hand, the ROS-sensitive AsA-deficient *vtc2* mutants have unusually low AsA levels (10–20% of the WT). Despite the fact that *VTC5* expression is 100–1000 times lower than that of *VTC2*, the double knockout of *vtc2*/*vtc5* can cause the cessation of seedling growth, damage that can be reversed when plants are fed with AsA itself or l-galactose [20,21], suggesting the requirement of both genes for AsA biosynthesis. As both *VTC2* and *VTC5* transcript levels are not induced in response to H_2_O_2_ or other oxidative stress, indicating that the signal is not ROS-related, *VTC2* is suggested to regulate the pathway by feedback inhibition via reduced translation [5]. Indeed, *VTC2* can be regulated by a non-canonical upstream open reading frame (uORF), which encodes for a peptide that acts as an inhibitor of translation when AsA is highly abundant, or a stimulator of GGP translation when AsA is at low levels [26]. Recent studies on a cis-acting uORF in GGP1 highlighted its role in regulating redox homeostasis, normal plant development, and pollen fertility in tomato [27]. On the other hand, the partial AsA deficiency in *Arabidopsis vtc4* mutants, in combination with the fact that these mutants also contained low levels of *myo*-inositol reinforces the notion that this enzyme has probably a double activity on both l-galactose-1-P and d-myo-inositol 3-P [23].

As the last enzyme of the l-galactose pathway, l-galactono-1,4 lactone dehydrogenase (GLDH), is placed and active in the mitochondrion, l-galactono-1,4-lactone must be transferred from cytosol to the inner mitochondrion membrane, to produce AsA (Figure 1). An interesting note here is that this final reaction in the AsA biosynthetic pathway is catalyzed by a dehydrogenase via cytochrome c, and not by an oxidase, as in the animal pathway. Consequently, no H_2_O_2_ is released, and no side effects over the cell redox state occur [28], highlighting the significance of the interaction between AsA synthesis and energy metabolism via the respiratory electron chain [29], which is yet to be entirely clear.

Several alternative AsA biosynthetic pathways have been proposed in different plant species, including those related to d-galacturonic acid or *myo*-inositol (Figure 1), but the overall contribution of these routes on AsA accumulation is highly debatable and seems to be predominantly species- and stage-dependent. For example, the d-galacturonic acid pathway via d-galacturonate reductase (GalUR) may supplement the AsA pool size in certain species such as strawberry [30], orange [31], apple [32], grape [33], and rose [34], or at specific developmental stages, for example, ripe tomato fruit [35]. On the other hand, the alternative route derived from *myo*-inositol, which is predominantly involved in hexose, starch, and pectin metabolism, rather than in AsA biosynthesis, seems to be less clear, and unlikely to significantly fine-tune AsA accumulation [14,36,37]. Notwithstanding, as the AsA pathway through this way is considerably shorter than through the l-galactose pathway, it may complement the predominant biosynthetic route particularly in fruit tissues under stress conditions [33].

Beyond its biosynthesis, AsA can undergo regeneration through the so-called ascorbate-glutathione cycle (Figure 1), as initially proposed by Foyer and Haliwell [38], to preserve redox cellular homeostasis. Being a strong antioxidant molecule, AsA can receive electrons from several free radicals. In this process, AsA undergoes enzymatic recycling from its oxidized forms, monodehydroascorbate (MDHA) and dehydroascorbate (DHA), with the activities of GSH reductase (GR), dehydroascorbate reductase (DHAR), and monodehydroascorbate reductase (MDHAR) [3,39]. If it is unable to be regenerated, DHA endures irreversible degradation, producing a wide range of products, including oxalic, tartaric, and threonic acids, depending on species. Tartaric acid formed in the cytoplasm is particularly important for the fruit quality of grapes [40,41]. In the apoplast, DHA degradation involves a mix of enzymatic and non-enzymatic reactions, including the oxidation of 4-*O*-oxalyl l-threonate and the hydrolysis of 2,3-l-diketogulonate, which is a harmful molecule producing H_2_O_2_ non-enzymatically [5,42]. Therefore, the AsA pool size should be finely tuned by efficient recycling to provide direct protection against free radicals. In tomato fruits, DHA degradation mainly generates oxalic and threonic acid [43], suggesting that DHA oxidation prevails over DHA hydrolysis. In the apoplast, AsA can also undergo oxidation via ascorbate oxidase (AO), an enzyme with vital roles in oxygen removal and signaling to protect redox homeostasis in the extracellular matrix [44,45]. In tomatoes, AO activity has been linked with sugar metabolism, with RNAi lines exerting improved yield stability and higher sucrose and hexose contents [46].

## 3. Ascorbic Acid Accumulation and Metabolism in Tomatoes

Considering the broad range of functions of AsA in fruit tissues, it is of great importance to understand why this molecule accumulates at high concentrations in some particular species and cultivars, and not in some others, but also what is the functional significance of this variability. As a general rule, concentrations in fruit AsA content tend to be high in wild accessions and lower in cultivated species due to the domestication process [47]. In tomato fruit, concentrations may range from 10 to 40 mg 100 g^−1^ in cultivated species [14,47,48,49], whilst wild accessions, such as *S. pennellii* or *S. pimpinellifolium*, have nearly five-times and three-times higher levels, respectively [50]. The range of natural diversity found within the tomato germplasm can be considered as moderate, compared to other fruit species, such as strawberry, apple, and kiwifruit (Table 1). Since the dawn of plant breeding, cultivar selection was based on a few key genes exerting a large phenotypic effect on desirable traits such as fruit size, shape, and color, as well as disease resistance [51]. As tomatoes are grown under more controlled environmental conditions, devoid of stress, it is evident that a low selective pressure to keep alleles conferring enhanced AsA levels may have taken place during the domestication process [6,48]. Tomato genetic resources include wild, landraces (heirlooms and local cultivars), modern cultivars, and breeding populations, offering untapped genetic and phenotypic diversity, with over 20,000 accessions being kept in gene banks over the world [51].

As AsA content is under the fine regulation of multiple genomic regions, the existence of great intra-species diversity within the tomato germplasm is of paramount importance for breeding not only due to its high nutritional value but also because of its potential implication in plant abiotic stress tolerance. Towards this end, the use of different populations derived from crosses between modern varieties and wild species serves as a valuable resource for genomic regions regulating AsA content in the tomato fruit. Stable chromosomic regions targeting chromosomes (Chr) 2, 8, 9, 10, and 12 have been implicated in AsA accumulation in Quantitative Trait Locus (QTL) studies [50,55]. For instance, within the stable QTL regions of Chr9, orthologue copies of key genes of the AsA metabolic pathways such as *GME*, *GMP*, and *MDHAR* were detected. Further studies confirmed the role of MDHAR in governing the tomato fruit AsA pool through the ripening process [14], or for extending postharvest life [55], whilst Genome Wide Association Studies (GWAS) revealed several key single-nucleotide polymorphisms (SNPs) associated with *MDHAR* [56].

Introgression lines (ILs) derived from the cross between the wild *S. pennellii* and M82 cultivar can serve as a valuable mapping source for QTL analysis [57]. In particular, a sub-line of the region 12-4 (IL 12-4-SL) harboring a QTL for enhanced AsA content at the bottom of chromosome 12 has been previously identified [58]. The accumulation of AsA in this line is approximately 40% higher compared to M82, probably as a result of the enhanced metabolic flux through the d-galacturonate pathway, which is driven by cell wall and pectin degradation triggered by ethylene during the ripening process [58,59]. Additionally, in the high AsA content IL, the expression of an orthologue of ascorbate peroxidase (*APX*) was significantly down-regulated, suggesting that AsA degradation may also contribute to the enhancement of the AsA pool in this line. Supporting this notion, silencing of *AO* caused a significant increase in AsA content in ripe melon [44]. Several genes involved in pectin degradation such as polygalacturonase (*PG*), pectinmethylesterase (*PME*), and UDP-d-glucuronic-acid-4-epimerase were also found to enhance the AsA pool via the alternative d-galacturonate pathway [60]. Recently, a group of genes related to sugar and hormone pathways were mapped within, and out of, the introgressed region of IL 12-4, and thus considered candidate genes regulating the AsA pool in the fruit of the sub-line [61]. Recombinant inbred lines have been also employed to identify key genes governing AsA levels in tomato fruits, evidencing the co-regulation of AsA and hormone metabolism [62].

Nonetheless, apart from the profound genetic differences in the AsA pool between cultivars, remarkable differences can be also attributed to different environmental (presumably light and temperature) and growing conditions, as well as ripening stage at harvest and postharvest handling [2,63]. Among them, the ripening stage at harvest is probably the key regulatory factor in climacteric species such as tomato, as ripening is thought to be an oxidative process influencing both antioxidant machinery and senescence-related processes such as cell-wall loosening [64]. Despite the inconsistency of findings related to AsA accumulation patterns in tomatoes, the general consensus is that young fruits at early developmental stages usually exert higher AsA biosynthetic capacities than mature ones, to support cell division and expansion. By contrast, as ripening progresses, AsA accumulation can be either due to increased recycling and/or decreased breakdown [14,47], or due to enhanced biosynthesis via *GGP* and *GPP*, and also correlated with increased respiration rates [13]. Using weighted gene correlation network analysis (WGCNA), others reported a lack of correlation between AsA biosynthetic genes and AsA accumulation rate in ripening tomato fruits [65], indicating the cultivar-dependent manner of AsA regulation under different growing conditions. At the gene level, the changes in AsA accumulation during tomato ripening have been correlated with transcript levels of key genes of the l-galactose pathway such as *GGP*, *GPP* [13], or *GGP* [14], and of the recycling pathway, that is, *MDHAR*, but not with the expression of genes from the alternative biosynthetic pathways such as *myo*-inositol oxygenase (*MIOX*), suggesting that it may serve as a supplementary pathway to support biosynthesis via the main route. By contrast, other genes from the main biosynthetic pathway were found to be related only in early fruit development [13] and in co-expression analyses using RNA-Seq data [58]. The same study confirmed a putative role for the d-galacturonate reductase gene during the last stages of fruit ripening, in line with previous findings based on feeding experiments [35]. As for AsA degradation, *AO* transcript levels were high early in tomato fruit ripening and then declined [13]. Nevertheless, a WGCNA study between tomato genotypes with contrasting antioxidant levels revealed an *AO* ortholog (Solyc07g052230), which favors the accumulation of the reduced form of AsA during ripening by regulating AsA redox state in the apoplast [66]. Therefore, it is clear that these structural genes (and their isoforms) exhibit cultivar- and stage-dependent regulation.

## 4. The Role of Ascorbic Acid to Confer Abiotic Stress Tolerance in Tomato Plants

In plant tissues, probably due to its low energetic biosynthetic cost, its low toxicity compared to other antioxidant compounds, as well as to its ability to be easily recycled, AsA is highly abundant in nearly all subcellular compartments and the apoplast, as well as in both photosynthetic and non-photosynthetic tissues [2,16,67]. The ubiquity of AsA in plant tissues is, in turn, responsible for the remarkable diversity of its function in plants. Briefly, AsA participates in ROS detoxification either directly or via the AsA-GSH cycle, in plant development and hormone signaling, in cell cycle and cell expansion, in flowering, in seed germination and viability, in regenerating other antioxidants, in plant responses to abiotic stress and pathogen attack, in the cellular redox system, as well as an enzyme cofactor [4,6,29,39,48,67]. Given that light is considered the most important environmental regulator of AsA [20,68,69], its accumulation is usually higher in young leaves and fruits, rather than in photosynthetically non-active tissues such as roots [48]. In this regard, AsA has a vital role in governing plant responses to abiotic stress factors [70], which is further discussed.

### 4.1. AsA as a Key Molecule under Abiotic Stress

Abiotic stresses such as drought and salinity are within the main environmental challenges that limit the global productivity of major crops [71,72]. Plants receive a variety of environmental pressures from the ecosystem, such as water shortage, extremely high or low temperatures, excess salt, or toxic metals in the soil affecting their growth adversely. One of the most important reactions to abiotic stress is the production of ROS consisting of both free radicals (superoxide radicals, O_2_^−^, hydroxyl radical, OH^−^) and non-free radicals (hydrogen peroxide, H_2_O_2_, and singlet oxygen ^1^O_2_), which causes oxidative damage to cellular components, proteins, DNA, and lipids and degrades cell structures when in excess [73]. The accumulation of ROS causes various damages including the peroxidation of lipid membranes and the production of malondialdehyde, which destroys membrane integrity [16]. To detoxify ROS, plants have developed enzymatic and non-enzymatic antioxidant mechanisms, including the accumulation of AsA [8,74], as well as the activation of antioxidant enzymes [67].

Through its roles in cell division and expansion, in acting as an enzyme cofactor, in participating in photosynthetic apparatus and hormone biosynthesis, AsA can protect cells and tissues by detoxifying stress-induced ROS accumulation [74]. Furthermore, as AsA can directly donate electrons to tocopherol radicals, it contributes to the reduction of lipid peroxidation and membrane protection [8]. A broad number of studies demonstrated that abiotic stress induced the activity of the enzymes involved in the AsA-GSH cycle, such as APX, MDHAR, DHAR, and GR in many species [67]. AsA serves as a specific electron donor for APX in the conversion of H_2_O_2_ to H_2_O. In this reaction, APX has a high affinity for H_2_O_2_ scavenging, effectively removing this non-free radical even at low doses, orchestrating ROS signaling under oxidative stress. In contrast to the cellular AsA pool that remains largely at a reduced state even under stress exposure, a high accumulation of the AsA oxidized form is usually observed in the apoplast, providing a putative regulatory mechanism for plant growth and cell-wall loosening and lignification under stress [40,75,76,77,78]. Thus, through regulating the redox state in the extracellular matrix, AO serves as a modulator of both AsA and ROS accumulation in the apoplast [47,76], having important implications on signaling under unfavorable environments [45,76]. Additionally, AO has a vital role in auxin degradation in response to osmotic stress [79], highlighting its diverse roles in mitigating stress injury.

Similar to other plant species, tomato plants suffer from a wide range of environmental stress factors, with the most important being drought, salt, and high temperature/high light. Under these conditions, AsA levels alter dramatically. For example, drought stress significantly increases the accumulation of osmolytes such as proline and soluble sugars, and of antioxidants including AsA to protect them from oxidative damage [8,80]. Tomato can be classified as a moderate salt-sensitive crop, possessing a salinity threshold measured as electrical conductivity (EC) at 2.5 dS·m^−1^, with relative yield losses of 50% when the substrate salinity is approximately at EC 7.6 dS·m^−1^ [81]. Under salinity stress, AsA is an essential compound of non-enzymatic antioxidants in plants, functioning in plant growth and hormone signaling and playing especially critical roles in the fine control of ROS homeostasis to improve salt tolerance [3,82].

Despite the increased demand for AsA accumulation to alleviate oxidative damage, AsA biosynthesis can be differentially regulated depending on the variation and intensity of stress factors. In particular, transcript levels of *GalUR* were remarkably enhanced by salt and oxidative stresses in tomato leaves, whilst AsA levels were reduced [83]. In tomato fruits, only the expression of *GPP* was enhanced under wounding and cold stresses, whereas both *AO2* and *MDHAR2* transcripts were induced under cold and wounding [13]. An interesting note is that 48 h anoxia did not provoke any induction on AsA biosynthetic genes in tomato fruit except for *MIOX* in tomato fruit. However, post-anoxic conditions caused an AsA increase accompanied by the induction of most of the biosynthetic genes as well as *MDHAR1*, *MDHAR2*, the thylakoid-bound *APX*, *SOD*, and *GR,* implying the accumulation of ROS due to anoxia. Furthermore, modifications in fruit AsA levels under stress conditions can also alter genes related to hormone-signaling, therefore influencing both hormone biosynthesis and signal transduction [62].

### 4.2. The Role of Ethylene in Regulating AsA Pathway in Fruits and Plants Exposed to Stress Factors

A close link between ethylene metabolism and AsA accumulation has been previously suggested in tomato fruit [84,85]. Transcriptome studies revealed remarkable alterations in the transcript levels of ethylene-, cell-wall-, and pigment-related genes at the beginning of the ripening process [84,86,87] that could probably affect carbon flux to the AsA pool, depending on fruit demands. In this regard, the availability of a broad number of well-characterized mutants that fail to ripen normally enables the in-depth study of the putative role of ethylene in AsA accumulation of tomato fruits through the course of ripening [85,86]. Among them, the role of the ethylene-insensitive never-ripe mutation that blocks the ethylene perception has been most widely studied. Fruits from nearly isogenic lines homozygous for the *Nr* mutation generally show a delayed onset of ripening, accompanied by poor coloration and marginally softening as ripening progresses, as well as enhanced AsA accumulation [84]. This suggests a possible link between AsA accumulation and ethylene perception, probably mediated by the delayed up-regulation of cell-wall-related genes such as *PG*, even though the exact mode of action remains obscure. Our previous findings demonstrated that AsA accumulation at the later stages of ripening can be affected by an interruption in the signal transduction of ethylene-mediated ripening pathways through the *Nr* locus [87]. Interestingly, the location of the *Nr* locus (Solyc09g075440) on the bottom of chr9 gives further support for this association, as it co-locates within the stable QTLs for fruit AsA concentrations in tomato [50].

The AP2/ERF (APETALA2/ethylene-responsive element binding factors) transcription factors are a large group of factors, present mainly in plants. Placed last in the ethylene signaling pathway [88], they regulate many developmental and physiological processes and participate in responsive mechanisms to various stresses. The AP2/ERF family is divided into four major subfamilies: DREB (Dehydration Responsive Element-Binding protein), ERF (Ethylene-Responsive-Element-Binding protein), AP2 (APETALA2), and RAV (Related to ABI3/VP), whereas few unclassified factors, consist of the Soloists group [89]. Several transcription factors, and specifically those related to ethylene signaling or perception, have been proposed to modulate the AsA content of plants exposed to oxidative stress or during plant growth, through regulation of expression patterns of genes implicated in the AsA biosynthetic/recycling pathway. In tobacco seedlings over-expressing a tomato ERF protein namely *JERF3*, an ethylene-induced gene, as well as cytosolic *APX1*, chloroplastic *APX2*, and glutathione peroxidase (*GPX*), all considered to use H_2_O_2_ as an electron acceptor, were up-regulated three to eight times compared to wild type plants [90]. Transcriptional activation of ROS-related genes by *JERF3* resulted in a decrease in accumulation of ROS and induced tolerance to drought, salt, and freezing. Another tomato *ERF* transcription factor, named *TERF1,* may also regulate ROS production or scavenging [91]. After being exposed to ethylene gas, the tobacco *TERF1*-expressing seedlings showed much lower superoxide and H_2_O_2_ content in relation to wild type. Not only genes catalyzing oxidative reactions, such as *GPX* but also *GMP* from the biosynthetic pathway, seemed to be transcriptionally induced under stress conditions. Therefore, *TERF1* renders stress tolerance of tobacco seedlings to H_2_O_2_. Those results suggest that *TERF1* is an ethylene inducible factor regulating ROS scavenging during stress responses. In *Arabidopsis*, it has been recently demonstrated that ethylene and ABA antagonistically orchestrate AsA biosynthesis and ROS accumulation in response to abiotic stress factors, via ETHYLENE-INSENSITIVE3 (EIN3) and ABA INSENSITIVE4 (ABI4) transcriptional cascade [92]. Further studies are required to unravel this complex transcriptional cascade of AsA regulation in tomatoes during development, fruit ripening, and in response to environmental stimuli.

### 4.3. The Role of AsA in Mitigating Post-Harvest Losses in Tomato Fruits

During postharvest life, fruits and vegetables could be exposed to different abiotic stresses [93] such as wounding, phytohormones, temperature, ultraviolet light, storage atmosphere gas composition, and dehydration [94,95]. In tomato, these losses have been estimated from 10 to 40% [96]. Therefore, suitable postharvest handling is essential to maintain fruit quality and in parallel to extend their marketable period during wholesale until their consumption from consumers [4]. Various treatments have been applied to provide postharvest abiotic stress resistance in fruits and vegetables, but with limited success since tissue response to abiotic stress is a highly complicated system of metabolic processes [97]. Moreover, stress factors could accelerate phytochemical damage in horticultural produce, with loss of AsA as the most sensitive indicator of stress exposure [13,98,99,100].

**Figure 2:**
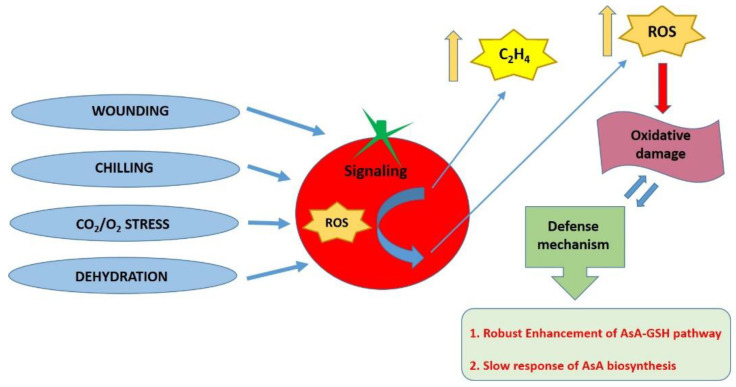
Schematic diagram of postharvest abiotic stress of tomato fruit and oxidative stress response. Fruit exposure to postharvest abiotic stress conditions such as wounding, chilling temperature, CO_2_/O_2_ injury, and dehydration, provoke a significant increase in ethylene (C_2_H_4_) and reactive oxygen species (ROS), which in low concentration serve as signaling molecules to regulate biological and physiological processes, whereas in high concentration can cause important damage to molecules and cell structure. Fruits organize an elaborate antioxidant network system as a defense mechanism, with AsA playing an important role especially by the robust enhancement of the AsA-GSH pathway, accompanied by a slower response of AsA biosynthesis.

Practically, to extend the marketability of tomatoes, it is common to harvest fruits at unripe mature stages and store them at low temperatures increasing the risk of chilling injury disorder [101,102], resulting in non-uniform ripening, the appearance of surface cracks, rapid loss of firmness, increased respiration rates, and higher water loss [103,104]. In tomato fruit exposed to chilling temperatures, scavenging of ROS indicates a complex network of molecules and enzymes as a part of an antioxidant response mechanism, correlated to fruit shelf-life [105]. During postharvest storage, the initial content of AsA at harvest is critical for the function of the recycling pathway under stress conditions where reduced AsA is oxidized into the unstable MDHA which dissociates into AsA and DHA (Figure 2) [55]. In tomato, MDHAR has been found to act as the key enzyme in regulating the AsA recycling process under chilling temperature storage [55,106]. Additionally, AsA levels during storage at chilling temperature could be altered because of APX activity through AsA oxidation [13]. Further study confirms that *APX* gene expression is up-regulated during chilling stress in tomato fruit [107]. Furthermore, storage of tomato fruit at low temperatures can lead to an increase in GR activity [105,108]. In cherry tomatoes, most of the genes involved in AsA biosynthesis such as *GME* and *GalDH*, as well as isoenzymes of APX, MDHAR, DHAR, and GR, were enhanced at chilling temperature storage [108]. On the contrary, a short duration of cold storage enhances only *GPP* expression [13] suggesting that AsA biosynthetic pathways do not react rapidly under cold stress conditions [108]. Recently, treatments with methyl jasmonate have been proposed as an efficient strategy to regulate tomato post-harvest quality and aroma, by inhibiting ROS accumulation through controlling the AsA-GSH cycle [109].

All the above suggest a target point towards a better understanding of mechanisms controlling AsA accumulation/degradation and of its role in the postharvest performance of tomato. Recent advances in sequencing technology including RNA-sequencing provide useful tools to approach this important issue [110]. This, in combination with the exploration of tomato biodiversity in terms of better postharvest characteristics and richer AsA content, will put forth the basis for a better understanding of the factors controlling tomato keeping quality [111].

### 4.4. Transgenic Efforts of AsA Manipulation towards Abiotic Stress Tolerance

Apart from the obvious effect on AsA accumulation, overexpression of a wide range of AsA-regulatory genes could also enhance the tolerance of transgenic tomato plants to various abiotic stresses. For example, overexpressing a chloroplastic *MDHAR* in tomato plants simultaneously elevates leaf AsA levels and improves plant tolerance to temperature and methyl viologen-induced oxidative stresses [112]. The efficient regeneration of AsA seems to be able to remove the ROS and protect the photosynthetic apparatus by alleviating the photoinhibition of the photosystem under these stresses. In fact, transgenic plants showed a lower level of H_2_O_2_ generation, as well as a higher net photosynthetic rate and maximal photochemical efficiency, under low- and high-temperature stresses. In tomato plants, the *AdBiL* gene exerts an essential role in maintaining cellular ROS and reactive nitrogen species [113]. Overexpressing lines accumulate less H_2_O_2_ and O^−2^ are coupled with lower NO and SNOs compared to untransformed plants under chilling stress. This implies a possible physiological role of *AdBiL* in the activation of the key enzymes of the AsA-GSH pathway, which may have potential implications in developing chilling-tolerant crop varieties through genetic manipulation. However, in the same study, biosynthetic genes are not influenced by cold stress, except for *GPP* whose transcript levels accumulated at high levels starting at 3 h after exposure to 4 °C. This may also indicate that when increased AsA levels are required, the cell compensates by increasing the *GPP* transcript levels for more AsA production [13].

Transgenic tomato plants over-expressing *SlGME1* and *SlGME2* have enhanced stress tolerance based on less chlorophyll content loss and membrane-lipid peroxidation under methyl viologen (paraquat) stress, higher survival rate under cold stress, and significantly higher seed germination rate, fresh weight, and root length under salt stress [114]. Transgenic tomato plants overexpressing *GalUR* contain higher levels of AsA and are more tolerant to abiotic stresses induced by methyl viologen, NaCl, or mannitol than non-transformed plants [115]. Under salt stress of less than 200 mM NaCl, transgenic plants can survive, in contrast with control plants being unable to survive under such conditions. Finally, when four key biosynthetic genes, *GME*, *GMP*, *GGP*, and *GPP*, were pyramided in tomato by conventional hybridization, transgenic lines exhibited increased AsA content, along with enhanced light response, stress tolerance after 75 μM methyl viologen application, and AsA transport capacity [116].

Rather than being the rate-limiting step in enhancing AsA accumulation through recycling, enhanced cytosolic or chloroplastic *MDHAR* expression has been associated with improved tolerance to abiotic stress factors in tomatoes in a light-dependent mechanism [55,112,117]. Additionally, plants with reduced MDHAR activity also showed an arrest in growth and yield, as well as reduced fruit size and sugar content [43]. On the other hand, overexpressing *DHAR* resulted in a moderate increase in the AsA levels of tomato leaves [118,119] or fruits [118,120], as well as better tolerance to abiotic stresses such as salt [115,116], or temperature [119]. Silencing of *AO* (Solyc04g054690) in cherry tomato lines led to increased AsA, lycopene, and carotene contents in the fruits, and further improved plant growth parameters, fruit quality, and total yield per plant under salinity stress [121]. Similar results of *AO*-silencing-mediated tolerance were obtained against drought stress [46].

Among novel regulatory genes and transcription factors controlling AsA-mediated responses of tomatoes, overexpression of the tomato basic helix-loop-helix 59 (bHLH59) improved tolerance to methyl viologen induced stress [122], while overexpression of the regulatory factor *SlZF3* encoding a Cys2/His2-type zinc-finger protein with an EAR repression domain improved salt tolerance by enhancing the ROS-scavenging ability of the *SlZF3*-overexpressing plants [123]. NFYAs belong to the NFY (Nuclear Factor Y, or CCAAT-binding factor) complex, being abundant within the plant kingdom [124]. Transgenic tomato lines overexpressing SlNFYA10 showed enhanced sensitivity to oxidative stress [125].

## 5. Modern Technologies for Ascorbic Acid Biofortification in Tomatoes

The extensive functional characterization of all the key intermediate steps of the main AsA metabolic pathways for over 20 years now, enabled AsA biofortification in several crop species, including tomato. Unraveling the regulation of AsA accumulation can have a clear positive effect toward achieving a vitC-rich human diet, improved postharvest shelf-life, and increased (a)biotic stress tolerance in bio-fortified plants [8]. In tomatoes, several biotechnological strategies have been employed to control AsA levels, including the overexpression or downregulation of biosynthetic, recycling genes, or other regulatory factors, summarized in Table 2. However, so far, efforts to enhance AsA contents in tomatoes had rather limited success [3,4], presumably for two main reasons. One being the connection of AsA to energy metabolism and oxidative stress, which are both strongly affected by environmental stimuli [126], exerting a large epigenetic effect on the AsA pool, which has been under-estimated. The second being the relatively narrow genetic variability within the cultivated tomato germplasm as a result of the high selective pressure for traits related to yield or fruit morphology rather than nutritional value, which witnesses moderate potential for genetic improvement and breeding for this trait. Other approaches for AsA biofortification include the use of major AsA-associated Quantitative Trait Locus (QTL) in breeding, the translational modulation of key genes such as *GPP*, modern biotechnological methods such as genome editing, as well as the multigenic co-expression approach by pyramiding structural genes [127]. In light of these findings, to promote AsA accumulation in tomato plants and fruits beyond the current level, attention has been shifted towards intervening in multigene expression, as well as manipulating components of other, seemingly unrelated, networks such as transcription and regulatory factors.

### 5.1. Biofortification through Enhancing Ascorbate Biosynthesis

Over the last decades, a broad number of successful and less successful biotechnological approaches have tried to enhance AsA biosynthesis in tomato plants overcoming specific rate-limiting steps of the l-galactose pathway [3,8,141]. In spite of the fact that the early, non-specific for AsA synthesis, genes of the l-galactose pathway have not been employed in tomato plants, several structural genes from the following steps such as *GMP* [128,129], *GME* [18,114], *GGP* [126,130], and *GLDH* [131], have been successfully overexpressed or down-regulated, affecting the AsA leaf and fruit pool size to different extents (Table 2). In line with the fact that *GMP* has not been correlated with AsA levels in ripening tomatoes [13,14], its overexpression resulted in a moderate (1.5- to 1.7-fold) [126] or limited (up to 1.5-fold) [129] increase in AsA content in the leaves and fruits, respectively. Similarly, modifications of *GME* expression had a relatively limited effect on AsA content in either leaf or fruit tissues in tomatoes [18,114].

Several lines of evidence reinforce the initial hypothesis that manipulation of *GGP*, the enzyme that catalyzes the first committed step of the main biosynthetic pathway, could serve as a valuable strategy to enhance AsA levels in several crop species, including tomato, as its transcripts levels significantly correlated with AsA changes [14]. Indeed, overexpression of the kiwi *GGP* has led to an up to six-fold increase in AsA content in tomato fruit, and less, but still significant, in strawberry (two-fold), or potato (three-fold) [126]. This modification of *GGP* expression in tomato fruit also resulted in the loss of seeds and of the jelly of locule tissue, highlighting the putative involvement of AsA in inhibiting seed formation, although the exact mode of action is still unclear. Overexpression of *GGP* in a fruit-specific manner, resulted in a three-fold increase of AsA in ripe tomato fruit, whereas a similar approach for *GPP* did not result in a similar increase of fruit AsA (Kanellis et al., unpublished data). More recently, using combined forward genetics with mapping-by-sequencing approaches, the impaired pollen fertility that resulted in seedless tomato fruits has been related to the over-accumulation of AsA (up to 5-fold WT level) in AsA-enriched mutants [27]. In a less successful effort, Wang et al. [130] achieved a nearly 50% decrease in leaf AsA content, by down-regulating a tomato gene coding *GGP*. Interestingly, increased co-expression of both *GGP* and *GME* in *Arabidopsis*, resulted in an up to seven-fold increase in leaf AsA [26,142], indicating that these two genes operate synergistically to govern leaf AsA pool size [26,126,142,143]. This finding is consistent with the co-regulation of the transcript levels of *GGP* and *GME*, at least in tomatoes. In particular, overexpression of *GME* significantly reduced *GGP* transcripts [114], repression of *GGP* increased *GME* transcripts [130], and diminution of *GME* enhanced *GGP* transcripts in the transgenic lines [18], probably in an effort to maintain a stable AsA pool size.

A few years ago, Laing and co-authors [26] shed some further light on the feedback regulation of AsA levels via suppression of *GGP* translation under high AsA levels, which probably explains the relatively limited success in single transgenic interventions so far. The authors demonstrated that in a wide range of species including tomato, the 5′-untranslated region (UTR) of *GGP* contains a highly-conserved upstream open reading frame (uORF), that encodes a peptide that can inhibit its translation [26,144]. According to this model, when AsA accumulation is enhanced, the uORF is translated and inhibits *GGP* translation, whilst the uORF is disabled and *GGP* is translated when AsA levels are low. Considering the ubiquity of this uORF in *GGP* genes from mosses to angiosperms, it is evident that regulation of *GGP* mRNA translation without the need for gene transcription modification points at an attractive biotechnological target to adjust the AsA pool in plant tissues in response to altering environmental conditions that may need to bypass transcriptional regulation [143].

Notwithstanding the fact that *GPP* transcript levels have been correlated with AsA content in ripening tomatoes or in response to ethylene, wounding, cold, and post-anoxic conditions [13], it is unlikely that this step alone serves as the key rate-limiting factor for AsA biosynthesis, at least in tomato fruit. Consistently, overexpressing *GPP* in tomato resulted in a 1-7-fold increase in leaf AsA content, but no change in fruit AsA content [116]. However, when *GGP* and *GPP* were overexpressed simultaneously in *Arabidopsis*, a four-fold change in leaf AsA levels was recorded [135], highlighting the importance of the co-expression of structural genes to ensure the meaningful boost in the flux of the pathway. In this regard, tomato hybrids resulting from separate transgenic lines expressing the *GGP* and *GPP* genes in a fruit-specific manner did not exhibit increased levels of fruit AsA contents (Kanellis et al., unpublished data). As for the last two enzymes of the l-galactose pathway, *GalDH* and *GLDH*, none of them were found to exert a noteworthy effect on AsA levels of tomato tissues [131] exerting nearly null fold change compared to WT plants, while the effect of these genes may be more pronounced in other species, including rice [145,146], *Arabidopsis* [135], and tobacco [147].

Gene pyramiding is a classical method in plant breeding according to which desirable genes are assembled into a single genotype [148], which has gained considerable attention in order to improve the synthesis of certain metabolites, involved in carotenoid [149] and anthocyanin accumulation [150]. However, the application of gene pyramiding for AsA-related genes was not conducted until recently [116]. According to this study, AsA biofortification in tomato was achieved by pyramiding *GMP* × *GME*, *GGP* × *GPP*, and *GMP* × *GME* × *GGP* × *GPP* by the conventional crossing of individual transgenic lines. Results showed that the increase in AsA levels was comparable between single-gene transgenic lines and pyramiding-lines, clearly demonstrating that AsA cannot be enhanced beyond a level due to the feedback inhibition loop of AsA accumulation and homeostasis. Further studies are required to determine AsA accumulation by pyramiding genes from different regulatory pathways, to avoid the side-effects from feedback inhibition occurring when co-expressing genes from single metabolic routes.

Among the alternative biosynthetic routes for AsA biosynthesis, the one through d-galacturonic acid, which has a dual role involved in both cell wall pectins and AsA biosynthesis, has given the most promising results in tomato fruits. In particular, overexpression of *GalUR* from strawberry resulted in a two-fold increase in AsA levels of hairy roots [132], 1.2- to 2.5-fold change in fruits [115,133,134], and 1.3- to 1.6-fold change in leaves [133,134] (Table 2). Regarding the gulose biosynthetic route from the animal-like pathway, overexpressing the rat *GuLDH/GLOase* showed moderate AsA levels in tomato fruits [151], similar to potato tubers [152], despite the encouraging findings in other species such as tobacco (seven-fold increase) or lettuce (four-fold increase) [153]. The contribution of the d-glucoronate pathway or *myo*-inositol pathway to AsA biosynthesis is highly controversial in plant tissues, although it can affect carbon incorporation into the cell wall [15,36]. Overexpression of *Arabidopsis myo*-inositol oxygenase (*AtMIOX*), the enzyme catalyzing the conversion of *myo*-inositol to d-glucuronate, increased AsA contents 1.4-fold in green fruits [128]. Even if the transgenic efforts to enhance the tomato AsA pool size using genes from the alternative routes have had limited success so far, the fact that these pathways are shorter than the main biosynthetic route support the notion that they may have a complementary role especially under adverse growing conditions [33].

### 5.2. Biofortification through Improving Ascorbate Recycling or Repressing Oxidation

Apart from enhancing biosynthesis, AsA biofortification can be achieved by manipulating genes responsible for the regeneration of AsA from its oxidized forms through the AsA-GSH cycle, that is, *MDHAR*, and *DHAR*, in order to preserve cellular AsA homeostasis [3,39]. Several reports have demonstrated that this strategy can be a very efficient method to engineer AsA content in several crop species, as it can also provide improved tolerance under unfavorable growth [55,154] or storage conditions [15]. The vital role of *MDHAR* in governing tomato fruit AsA levels has been corroborated by analyzing both QTLs [50,55,56] and expression profiles during the ripening process [13,14]. Nonetheless, overexpression of the cytosolic *MDHAR* had a negative effect on AsA accumulation in tomato leaves, whilst lines with silencing *MDHAR* displayed enhanced AsA levels in both leaves and orange fruits [48]. Similar results were obtained by Haroldsen and co-authors [120], while Li et al. [112] reported a moderate increase in the leaf AsA content. On the other hand, when overexpressing DHAR in tomato plants, a moderate increase in AsA levels was recorded in both leaves [118,119], and fruits [118,120].

Within the cell, ROS scavenging and detoxification is predominantly carried out by APX, which detoxifies H_2_O_2_ and forms MDHA [155], whereas, in the apoplast, AO uses AsA to maintain an oxidizing environment generating DHA as a by-product [44,46,76,78,156]. Conceivably, the oxidation of the AsA pool by cellular APX or the apoplastic AO is unavoidable due to the pivotal role of AsA in ROS detoxification. Efforts to manipulate *APX* expression in plant species are rather limited, as APX is encoded by a vast number of isoforms with different cellular localizations that are responsible for the maintenance of cellular homeostasis [127]. On the other hand, blocking AsA catabolism via down-regulating *AO* expression has gained more attention [44,46,79], as this strategy can have a tremendous impact on plant oxidative responses to stress. In tomato, efforts to manipulate different AO isoforms yielded contradictory effects on AsA contents, that were found to be unaltered [44] or increased [121,157] due to the prevention of AsA breakdown. Nonetheless, silencing *AO* has led to improved oxidative stress tolerance in tobacco and *Arabidopsis* [158], whereas in tomatoes, it resulted in better plant growth features and fruit yield under salinity stress, and higher photosynthesis under drought stress [121]. In melon fruit, silencing *AO* has led to a spectacular increase in the apoplastic AsA content with the simultaneous induction of key biosynthetic and recycling genes, as well as increased ethylene production and decreased fruit size [44]. These novel findings for melon support the role of *AO* in fruit growth, at least in *Cucurbitaceae*, and further suggest the potential of AsA enhancement through this unexpected route.

### 5.3. Biofortification through Novel Regulatory Genes and Transcription Factors

As the manipulation of structural genes from the AsA biosynthetic and recycling pathways has only had limited success so far (Table 2), it is becoming evident that other key components of AsA accumulation, including transcription factors and regulatory proteins, also co-exist in model and crop species. In tomatoes, RNA-Seq studies revealed that several transcription factors, including MYB, NAC, and ZIF, may control the expression of AsA-biosynthetic genes, and further correlate with fruit AsA content [159], whilst a weighted gene correlation network analysis study demonstrated that the AsA biosynthetic genes had weak connectivity to AsA accumulation in ripening tomatoes [65]. In light of this observation, overexpressing the *SlHZ24* transcription factor that binds the promoters of *GMP*, *GME*, and *GGP* in tomatoes, resulted in a 1.5-fold and 1.2-fold increase of leaf or fruit AsA content, respectively [136].

Similarly, the tomato *basic helix-loop-helix 59* (*bHLH59*) gene, that co-localizes with an AsA-related QTL, regulates the expression levels of several genes of the l-galactose pathway, such as *PMM*, *GMP*, and *GME* [122]. Overexpressing this gene increased the leaf AsA content 1.5-fold, while RNAi lines had only 65% of WT AsA levels. The effect of *bHLH59* on AsA accumulation is due to nucleotide differences in the promoter region of HLH59. Overexpression of the regulatory factor *SlZF3* significantly enhanced AsA content in tomato leaves (Table 2) [123]. *SlZF3* promotes the accumulation of AsA by preventing *GMP* degradation and ubiquitination through binding COP9 signalosome subunit 5B (CSN5B), a key component of the photomorphogenic COP9 signalosome, and thus inhibiting the binding of CSN5B to *GMP*.

The DNA-binding with one finger (Dof) proteins are plant-specific transcription factors with diverse biological functions, such as large gene families in most plant species, including tomato with 34 gene members [160]. The *SlDof22* factor has been demonstrated to negatively regulate AsA levels and transcription of AsA biosynthetic and recycling genes in tomato [137]. In particular, leaves and fruits of the *SlDof22* RNAi transgenic tomato lines had 1.3- and 1.6-fold higher AsA levels, respectively, compared to WT plants. Furthermore, the expression of *GGP*, *GalDH*, *GLDH*, *MDHAR*, cytosolic *APX,* and *GR* was induced in transgenic plants. Nonetheless, knockdown of the *SlDof22* gene also resulted in a decreased tolerance to salinity, which was associated with the downregulation of the *salt overly sensitive 1* gene.

In total, the tomato genome contains 43 NBS-LRR (NL) subfamily resistance proteins, whose function is largely unknown. Knock-down of *SlNL33* gene raised the AsA content of leaves and fruits 2.7- and 1.3-fold, respectively, in *SlNL33*-RNAi transgenic lines compared to WT plants [138]. The accumulation of AsA in the transgenic plants has been attributed to the high transcript levels of nearly all the structural AsA-related genes, such as *GMP*, *GME*, *GGP*, *GPP*, *GalDH*, *GLDH*, *MIOX*, *APX*, *MDHAR,* and *DHAR*. Although the exact mode of interaction between AsA metabolism and *NL33* is still unclear, the suppressed expression of *SlNL33* also promoted tolerance to methyl viologen and gray mold infection by *Botrytis cinerea*, probably via enhancing the ROS scavenging capacity through the AsA-GSH cycle.

The tomato CCAAT-box transcription factor (*SlNFYA10*) has been recently demonstrated to be the first CCAAT-binding factor negatively controlling AsA synthesis at multiple sites, as it can bind to the promoter of *GME* and *GGP1* [125]. Transgenic tomato lines overexpressing *SlNFYA10* contained significantly lower AsA contents in their leaves and fruits, as a result of the decreased abundance of both genes.

d-Arabinono-1,4-lactone oxidase (*ALO*), the yeast analog GLDH, catalyzes the conversion of d-arabinono-1,4-lactone to erythroascorbate [161]. Overexpressing *ALO* in tomato increased leaf and green fruit AsA contents by 1.5-fold and 1.25-fold, respectively, possibly by pulling carbon flux towards AsA biosynthesis, while AsA turnover was also induced as indicated by the high DHA levels, probably allowing feedback responsive regulation of AsA synthesis [128].

Malate dehydrogenase (MDH) is the enzyme responsible for the reversible reduction of oxaloacetate to malate. Antisense expression of a mitochondrial *MDH* in tomato has led to significant induction of AsA accumulation in leaves, and simultaneously, a repressed rate of flux through the TCA cycle, a reduced rate of respiration, and an increased photosynthetic rate in the transgenic plants compared to WT plants [139].

The tomato *High-Pigment-1* (*hp1*) mutants are functionally deficient in the negative regulator of UV-Damaged DNA-Binding Protein 1, exerting enhanced carotenoids [162] and flavonoids [163], and reduced AsA levels [140], compared to WT plants. The decreased AsA accumulation at various stages of fruit development and ripening has been attributed to the differential expression of several structural genes from the AsA biosynthetic and recycling pathways in the *hp-1* fruit. In particular, through the course of fruit development and ripening, the expression of *GMP*, *GME*, and *GPP* was higher in *hp-1* than in WT plants, while the expression of *MDHAR* was lower.

### 5.4. Biofortification through Genome Editing

The recent advances in genome editing technologies can serve as an alternative rapid transgene-free tool to develop tomatoes with increased AsA levels. The ability to generate specific modifications in the genome due to the targeted design of sequence-specific nucleases is one of the main advantages of genome editing compared to earlier mutational breeding strategies using transgene silencing and random insertion of T-DNAs [127]. Furthermore, as genome-edited plants do not contain any foreign DNA, they are unlikely to be subjected to regulatory oversight [141]. Regarding AsA content, it has been demonstrated that *GGP* contains an unusual but highly conserved uORF [26,143], that is preferentially translated when AsA is highly accumulated, thus reducing GGP protein abundance. Targeting this effective negative feedback loop that enhances AsA levels, the CRISPR/Cas9 genome editing system has been recently employed in *Arabidopsis,* lettuce, and tomato, by disrupting the *cis*-acting *GGP* uORF [144]. This efficient method of manipulating the translation of mRNA resulted in a 1.5-fold increase in AsA contents, and further improved stress tolerance to MV-induced oxidative stress in lettuce. More recently, AsA-enriched tomato mutants were generated by targeting the uORF of the *SlGGP1* coding sequence, linking high AsA levels with impaired floral development and pollen fertility, as well as seedless fruits [27]. This intriguing finding that links AsA and GSH with plant reproduction has been previously reported in tomato lines overexpressing *SlGGP1* [126]. Additionally, RNA-seq analysis of these CRISP-mutated lines confirmed that this particular uORF acts as a regulator of AsA synthesis and redox state to enable normal plant/organ development [27].

Other potential targets for genome editing in tomato include the *Arabidopsis* regulatory factors *AsA mannose pathway regulator 1* (*AMR1*), *CSN5B*, *CSN8*, *NL33*, that are able to enhance AsA levels when disrupted with T-DNA insertions or downregulated with RNAi [141]. The challenge of using genome editing technologies to enhance the AsA of crop species has phenomenal potential as it can minimize pleiotropic defects due to carbon reallocation in plant growth and fruit development occurring when modifying single genes from the AsA biosynthetic pathways [26].

## 6. Conclusions

Although the structural genes involved in AsA metabolic pathways have been well characterized in many plant species including tomato, several aspects of AsA regulation and its interaction with other metabolic pathways or hormones, especially under stress conditions, remain to be further investigated. Transgenic approaches towards increasing the expression of single AsA-metabolic genes are a common strategy to both enhance AsA contents and improve tolerance to a broad range of abiotic stress factors. More recently, the manipulation of AsA regulatory genes, or multiple gene pyramiding, has emerged as an alternative way to increase AsA contents of horticultural species. A broad number of studies reinforce the hypothesis that non-structural AsA-related genes such as transcription factors may be important in some species, tissues, or ripening stages, orchestrating AsA poll size at either the transcriptional or post-translational level. Nonetheless, no such efforts towards modifying cellular redox homeostasis come without any challenge. In this manner, fine-tuning of AsA accumulation and of its feedback regulation is necessary to minimize any pleiotropic effect on plant growth and development, as well as plant responses to the abiotic stress of tomatoes.

## Figures and Tables

**Table 1:** The AsA content in commonly consumed fruits.

Species	Fruit	AsA Content (mg/100 g FW)	Reference
*Malpighia emarginata*	Acerola	1190–2187	[52]
*Actinidia deliciosa*	Kiwifruit	29–120	[2,4]
*Fragaria* × *ananassa Duch.*	Strawberry	10–80	[2,4,6]
*Solanum lycopersicum*	Tomato (cultivated varieties)	6–23	[14,47]
*S. pennellii; S. pimpinellifolium*	Tomato (wild species)	6–50	[47]
*Cucumis melo* L.	Melon	10–35	[2,6,7]
*Malus domestica Borkh.*	Apple (cultivated varieties)	1–13	[53,54]
*Malus* spp.	Apple (wild varieties)	2–28	[53]

**Table 2:** Engineering strategies used to manipulate AsA accumulation in tomatoes.

Pathway	Gene Name	Gene Code	Strategy	Gene Source	Tissue	Maximum AsA Fold-Increase	Reference
l-Galactose	GDP-mannose pyrophosphorylase	*GMP*	overexpression	Yeast	leaves	1.7	[128]
					fruits	1.5	
				Tomato	leaves	1–1.5	[129]
	GDP-d-mannose 3′5′-epimerase	*GME*	RNAi silencing	-	fruits	0.6–0.8	[18]
					leaves	0.2–0.5	
			overexpression	Tomato	leaves	1.2–1.4	[114]
					fruits	1.2–1.6	
	GDP-galactose-phosphorylase	*GGP*	overexpression	Kiwifruit	leaves	no change	[126]
					fruits	3–6	
			downregulation	Tomato	leaves	0.5–0.75	[130]
	l-galactose-1-phopshatase	*GPP*	overexpression	Tomato	leaves	1.7	[116]
					fruits	no change	
	l-galactono-1,4-lactone dehydrogenase	*GLDH*	RNAi silencing	-	leaves	0.9	[131]
					fruits	1.1–1.2	
	multiple modifications	*GME* × *GMP*	gene pyramiding	-	leaves	2	[116]
					fruits	1.25	
		*GGP* × *GPP*	gene pyramiding	-	leaves	1.3	
					fruits	no change	
		*GMP* × *GME* × *GGP* × *GPP*	gene pyramiding	-	leaves	2	
					fruits	1.25	
d-Galacturonate	d-galacturonate reductase	*GalUR*	overexpression	Strawberry	hairy roots	2	[132]
			overexpression	Strawberry	fruits	2.5	[115]
			overexpression	Strawberry	leaves	2	[133]
					fruits	1.6	
			overexpression	Strawberry	leaves	1.3	[134]
					fruits	1.2–1.4	
*Myo*-inositol and l-gulose	*myo*-inositol oxygenase	*MIOX*	overexpression	*Arabidopsis*	leaves	>0.75	[128]
					green fruits	1.4	
	l-gulono-1,4-lactone dehydrogenase	*GuLDH/GLOase*	overexpression	Rat	fruits	1.7	[135]
Recycling and breakdown	Monodehydroascorbate reductase	*MDHAR*	overexpression	Tomato	fruits	0.7	[120]
					leaves	no change	
			overexpression	Tomato	leaves	1.2	[118]
			overexpression	Tomato	leaves	0.7	[47]
					fruits	no change	
			RNAi silencing	-	leaves	1.2	
					fruits	1.2	
	Dehydroascorbate reductase	*DHAR*	overexpression	Tomato	fruits	1.6	[120]
					leaves	no change	
			overexpression	Potato	leaves	2	[118]
					fruits	1.4	
			overexpression	*Pyrus sinkiangensis*	leaves	1.5	[119]
	Ascorbate oxidase	*AO*	overexpression	Tomato	leaves	no change	[46]
					fruits	no change	
Transcriptor factors and other regulatory proteins	HD-Zip I Family Transcription Factor 24	*SlHZ24*	overexpression	Tomato	leaves	1.5	[136]
					fruits	1.2	
	basic helix-loop-helix 59	*SlbHLH59*	overexpression	Tomato	leaves	1.5	[122]
			RNAi silencing	-	leaves	0.65	
	Cys2/His2-type zinc-finger protein	*SlZF3*	overexpression	Tomato	leaves	2.1	[123]
			RNAi silencing	-	leaves	no change	
	DNA-binding with One Finger 22	*SlDOF22*	RNAi silencing	-	leaves	1.3	[137]
					fruits	1.6	
	NBS-LRR Resistant Protein	NBS-LRR 33	RNAi silencing	-	leaves	2.7	[138]
					fruits	1.3	
	Nuclear Factor Y or CCAAT-binding factor	*SlNFYA10*	overexpression	Tomato	leaves	0.65	[125]
				-	fruits	0.55	
	Arabinono-1,4-lactone oxidase	*ALO*	overexpression	Yeast	leaves	1.5	[128]
						1.25	
	Malate dehydrogenase	*MDH*	overexpression	Tomato	leaves	5.7	[139]
	High-pigment 1	*HP1*	overexpression	Tomato	fruits	0.7	[140]
